# *In Vitro* Digestion and Fecal Fermentation Characteristics of Extruded High Amylose Maize Starch with Different Moisture Contents

**DOI:** 10.3390/foods15111956

**Published:** 2026-06-01

**Authors:** Hongjie Zhang, Huifang Guo, Shujun Wang, Shaokang Wang

**Affiliations:** 1 State Key Laboratory of Food Nutrition and Safety, Tianjin University of Science & Technology, Tianjin 300457, China; 2 College of Food Science and Engineering, Tianjin University of Science & Technology, Tianjin 300457, China

**Keywords:** starch extrudates, different moisture contents, *in vitro* digestion and fermentation, elevated SCFA yields

## Abstract

In the present study, starch extrudates with varying moisture contents (30%, 40%, and 50%) were prepared by twin-screw extrusion; the morphology, structural order changes, *in vitro* digestion, and fecal fermentation characteristics were investigated. X-ray diffraction (XRD) and differential scanning calorimetry (DSC) analyses demonstrated the extrusion process severely disrupted the starch order, while the addition of water reduced this disruptive effect. The long-range ordered structure of starch extrudates gradually decreased with the increasing moisture contents, indicating the damage degree of starch extrudates increased with increasing moisture content. Compared to high maize 260 (NS), extruded starch (ES) and starch extrudates with different moisture contents (S-30, S-40, and S-50) exhibited a significantly higher hydrolysis rate, digestion extent, and fermentation rate, while no significant differences were shown among starch extrudates with different moisture contents. Interestingly, compared to ES, starch extrudates with high moisture contents (S-30, S-40, and S-50) exhibited significant higher levels of short-chain fatty acids (SCFAs). Pearson correlation analysis showed the yields of SCFAs were positively correlated with the content of V-type starch formed during extrusion. These findings provide a theoretical guidance for the design of starch-based extruded foods with varying moisture contents.

## 1. Introduction

Starch is the main source of carbohydrates in the human diet and accounts for a large portion of the daily dietary calorie intake [1]. It is formed of two glucan polymers, the essentially unbranched amylose, and the highly branched amylopectin [2,3]. Native starch has many advantages, including cost-effectiveness, easy availability, excellent renewability, and biodegradability. Nevertheless, it has functional limitations such as poor solubility in water, fast retrogradation, poor thermal properties, and low resistance to shear stress [4]. To overcome the shortcomings and endow native starch with properties that are needed for industrial applications, three kinds of modification approaches have been employed, including chemical modification, enzymatic modification, and physical modifications [5]. Among various modification methods, extrusion processing is a mature physical method that is an environment-friendly, highly adaptable, cost-effective, and energy-saving technology [6,7], which has been applied in the food industry for more than 80 years [8].

Different starch-based food products such as biscuits, pasta, snacks, bread crumbs, and breakfast cereals can be made by extrusion. The extrusion parameters such as the screw configuration, barrel temperatures, and water content are the key factors in the extrusion process and have a significant impact on the processability and product quality [9,10]. A low moisture content can increase the drag force at the die, and increase starch granules’ shear disintegration [11,12]. The lower moisture content has been reported to increase the starch digestibility in extruded buckwheat flour due to the increased starch gelatinization and molecular degradation [13]. The resistant starch (RS) content has also been reported to increase with the increasing moisture content in extruded flours. For example, Sumargo et al. [14] found that the RS content of the extruded brown rice and pinto bean flour mixture was increased while RDS decreased with the increase in moisture during the processing. Compared to the raw pastry wheat flour (0.38% RS), a higher moisture content (60%) and longer storage time (14 days) significantly increased the RS content by up to 11 times [15]. Similarly, the RS content in extruded rice flour [16,17] and phoenix flour [18] has been reported to increase with the increase in feed moisture, which may be attributed to the fact that high feed moisture increases the fluidity of starch molecules, leading to gelatinization and increases the tendency of starch retrogradation. However, contradictory results have also been reported. For instance, a higher RS content was observed in extruded oat flour under a limited moisture content. The authors believed that lower moisture created harsher conditions in the extruder due to the absence of the lubricating effect of water that leads to the increased fragmentation of the starch. The short starch chains generated at lower moisture re-associate after cooling due to the increased molecular mobility, promoting the formation of RS3 and thus leading to the high enzymatic resistance [19].

Apart from the digestibility, the moisture content also has an impact on the fermentation characteristics of starch-based foods. Rose et al. [20] found that a lower moisture content (15%) in extruded wheat bran flour produced higher SCFAs levels in comparison with higher moisture (30%) after 24 h of fecal fermentation. Lower moisture (15%) in extruded oat flour also have been reported to result in more butyrate production during the initial stage of *in vitro* fecal fermentation (0–8 h) [21]. Extruded rice bran insoluble fiber at different moisture contents (20%, 40%, and 60%) results in different yields of short-chain fatty acids; the extrudates with 40% moisture had the highest acetate, propionate, butyrate, and total SCFA after 48 h of fermentation [22]. As discussed above, most studies on the effects of the moisture content during screw extrusion have focused on flour; there is rare information regarding the changes in the starch structure and properties during extrusion under different moisture conditions.

Therefore, in the current study, we aim to understand the effects of the moisture content on the structural and functional properties of starch during the extrusion process. High amylose maize starch with different moisture contents (30%, 40%, and 50%) was used as raw material, with starch without added water as a reference, and then the extrudates were obtained by a twin-screw extruder. The changes in morphology, and long- and short-range ordered structures of the starch extrudates were characterized. On the basis of the molecular structure changes, the *in vitro* digestion kinetic profiles and *in vitro* fecal fermentation characteristics including gas and acid productions were examined.

## 2. Materials and Methods

### 2.1. Materials

High amylose maize starch (NS, amylose content of 61.1%), a common commercial high amylose maize starch after hydrothermal treatment, was obtained from Ingredion Co., Ltd. (Shanghai, China). *Alpha*-amylase from porcine pancreas (11 units/mg) and *para*-hydroxybenzoic acid hydrazide (PAHBAH) were purchased from Sigma-Aldrich Co., Ltd. (St. Louis, MO, USA). Other chemicals were of analytical grade.

### 2.2. Preparation of Starch Extrudates with Different Moisture Contents

High amylose maize starch (90 g, wet basis) was weighed in polypropylene bags; different amounts of distilled water was added to achieve the moisture contents of 30%, 40%, and 50% (wet basis). The bags were sealed and allowed to equilibrate for at least 2 h at room temperature before transferring into the SHJ-20 co-rotating twin-screw extruder equipped with a cylindrical diameter of 2 mm (Nanjing Jieya Extrusion Equipment Co., Ltd., Nanjing, China). The ratio of length to the diameter of the extruder is L/D ≈ 40. The barrel temperatures of the six heating zones were set at 85 °C and the screw speed was 180 rpm. In the extrusion process, the starch sample was fed into the hopper at a feed speed of 25 Hz, and the extrudates was obtained through a rectangular die with the length, width, and depth of 23 mm, 5.4 mm, and 37.3 mm, respectively. The extruded starch samples were dried in the oven at 40 °C, and grounded by a cryogenic mill (SPEX 6875, SPEX Sample Prep. Company, Metuchen, NJ, USA) for further analysis. High maize 260 (NS) and extruded starch without any water addition at the same processing condition (ES) were used as control. The obtained starch extrudates with different moisture contents are referred subsequently to as S-moisture content (S-30, S-40, and S-50).

### 2.3. Differential Scanning Calorimetry

Thermal properties of starch and starch extrudates were determined using a differential scanning calorimeter (DSC, 200 F3, Netzsch, Germany) equipped with a thermal analysis data station (NETZSCH Proteus Analysis, version 6.1.0). Starch samples (3 mg) were accurately weighted into an aluminum crucible, and 9 uL of deionized water was added to achieve a starch/water ratio of 1:3. The aluminum crucibles were sealed and allowed to equilibrate at room temperature overnight before the measurement. The samples were heated from 20 °C to 130 °C at a rate of 10 °C/min. An empty crucible was used as the reference. The thermal transition parameters (onset temperature T_o_, peak temperature T_p_, conclusion temperature T_c_, and enthalpy change ΔH) were obtained using the data analysis software of the instrument.

### 2.4. Scanning Electron Microscopy (SEM)

The morphology of starch and starch extrudates were observed using a scanning electron microscope (SU3800, Hitachi High Technology Co., Ltd., Tokyo, Japan) at an accelerating voltage of 5 kV. The samples were mounted on an aluminum stab using double-sided tape and sputter-coated with gold in a sputter coater (JEC-3000FC, JEOL Ltd., Akishima, Tokyo, Japan) prior to imaging.

### 2.5. X-Ray Diffraction (XRD)

The XRD analysis was preformed using an advance-wide-angle XRD (D8 Advance, Bruker, Karlsruhe, Germany) operating at 40 kV and 40 mA. The starch and starch extrudates were equilibrated over a saturated NaCl solution for 1 week before the measurement. The equilibrated samples were packed tightly in the quartz dish and scanned from 5° to 35° (2*θ*) at a rate of 2°/min and a step size of 0.02° [23]. The relative crystallinity was calculated using the DIFFRAC.EVA 4.2.1 software.

### 2.6. Fourier-Transform Infrared (FTIR) Spectroscopy

The FTIR spectra of starch and starch extrudates were analyzed by the Thermo Scientific Nicolet IS50 spectrometer (Thermo Fisher Scientific, Waltham, MA, USA). Samples (1 mg) and dried KBr powder (150 mg) were mixed evenly in an agate grinder. Then, the mixture was pressed in to transparent tablet and scanned from 4000 to 400 cm^−1^. Each spectrum was recorded against an air background at a resolution of 4 cm^−1^ with an accumulation of 64 scans. The obtained spectra were baseline-corrected automatically by OMNIC 8.0 and deconvoluted in the range of 1200–8000 cm^−1^ with a half bandwidth of 19 cm^−1^ and an enhancement factor of 1.9. The ratios of absorbances at 1047/1022 cm^−1^ was calculated to estimate the short-range ordered structure of the samples [24].

### 2.7. In Vitro Enzymatic Digestibility

The *in vitro* enzymatic digestibility of starch and starch extrudates were performed according to the procedure of Wang et al. [25] with some minor modifications. Gelatinized high amylose maize starch obtained by heating starch–water mixtures (85% water content) in an autoclave at 130 °C for 20 min was used as the reference. In brief, starch and starch extrudates (100 mg) were dispersed in 9 mL of 0.2 M sodium acetate buffer solution (pH 5.8), and 1 mL of α-amylase solution (60 U/mL) was added. The mixtures were incubated in a 37 °C water bath with magnetic stirring (260 rpm) for 2 h. Aliquots (0.1 mL) of the hydrolysate were withdrawn at intervals (10 min, 20 min, 30 min, 40 min, 60 min, 80 min, 100 min, 120 min, 150 min, 180 min, 210 min, and 240 min) and mixed with 0.9 mL of 0.3 M sodium carbonate solution to deactivate the enzymes. After centrifugation (13,000 rpm, 5 min), the maltose-equivalent reducing sugar content in the supernatant was determined using the PAHBAH assay, and the percentage of starch digestion was calculated using the standard curve of maltose solutions.

To gain a better mechanistic understanding of the enzymic digestion of starch and starch extrudates, the obtained digestograms were fitted to the first-order kinetics equation as follows:

$$C_{t}=C_{\infty }\;(1\;-\;e^{-kt})$$

where t is the digestion time (min), C_t_ is the amount of starch digestion at incubation time t, C_∞_ is the estimated amount of starch hydrolysis at the end of the reaction, and *k* represents the apparent digestion rate coefficient (min^−1^). The value of digestion rate coefficient (*k*) can be obtained from the slope of a linear least square (LOS) fit of a plot of ln (dC/dt) against t.

### 2.8. In Vitro Fecal Fermentation

*In vitro* batch fermentation was performed as previously described with minor modification [26]. In brief, starch and starch extrudates (50 mg) were weighted in triplicate into sterilized tubes for each time point. The carbonate-phosphate buffer was prepared and autoclaved at 121 °C for 20 min; then, cysteine (0.25 g/L of buffer) as the reducing agent was added under the CO_2_ atmosphere. The buffer and tubes with substrates were then transferred into the anaerobic chamber (HYX-II, CIMO Medical Instrument Manufacturing Co., Ltd., Shanghai, China) prior to incubation with human fecal microbiota.

Fresh fecal samples were collected from 3 healthy donors recruited from Tianjin University of Science and Technology (Tianjin, China). The donors were interviewed and signed the consent form before sample collection. All the donors are in good health with no gastrointestinal or metabolic diseases, have not been exposed to any antibiotics for at least 3 months, and have not consumed any prebiotics within past two weeks. The protocol for collecting human feces was approved by the Institutional Review Board of Tianjin University of Science and Technology (Approval Number: 2024093). Equal amounts of feces from each donor were mixed and diluted with sterilized buffer (feces: buffer = 1:3, *w*/*v*), followed by filtration through four cheesecloth layers to obtain the fecal slurry. Fecal slurry (1 mL) and buffer (4 mL) were then added to each bottle, sealed, and incubated at 37 °C in a water bath to start the fermentation. At the specific time points (4, 8, 12, and 24 h), gas production was measured using a graduated syringe. Then, the fermentation broth was centrifuged (13,000 rpm, 5 min), and the obtained supernatant was collected for pH and SCFAs determination. All the samples were stored at −80 °C before further analysis.

### 2.9. Analysis of Short-Chain Fatty Acids (SCFAs)

The concentrations of short-chain fatty acids were determined as previous described [27]. Briefly, 400 μL supernatant was mixed with 100 μL of a solution composed of 1.56 mg/mL copper sulfate, 5% meta-phosphoric acid, and 4-methylvaleric acid (internal standard). After filtration though a 0.45 μm filter membrane, 0.2 μL of the mixture was injected into the Gas Chromatography (GC-FID, Agilent Technologies, Inc. Santa Clara, CA, USA GC 2010 Plus, Shi-madzu, Kyoto, Japan) which was equipped with a fused silica capillary column (Zebron, ZB-FFAP, 30 m × 0.25 mm × 0.25 μm, J&W Scientific, Orangevale, CA, USA). The injector and detector temperatures were both set as 230 °C. The operating program was the same as described previously [28]. The SCFAs were identified based on the retention time, and the SCFA concentrations were calculated based on iso-caproic acid as an internal standard.

### 2.10. Statistical Analysis

All the experimental data reported were expressed as mean ± standard deviation (SD) of at least triplicate measurements. All data differences were judged by one-way analysis of variance (ANOVA) and Duncan’s multiple-range test, and Pearson correlation was analyzed by SPSS 26.0 Statistical Software Program (SPSS Inc., Chicago, IL, USA).

## 3. Results and Discussion

### 3.1. Thermal Properties of Starch and Starch Extrudates

**Figure 1:**
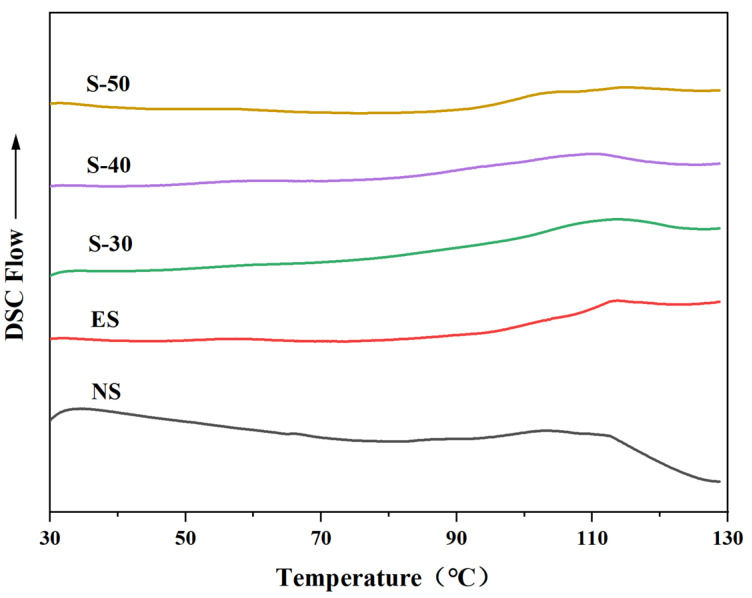
DSC curves of high maize 260 (NS) and starch extrudates with different moisture contents (ES, S-30, S-40, and S-50).

The DSC thermogram and the corresponding thermal transition parameters of NS and starch extrudates are presented in Figure 1 and Table 1. The onset (T_o_), peak (T_p_), and conclusion (T_c_) temperatures of NS were 88.0 °C, 101.9 °C, and 120.9 °C, respectively. After extrusion, the thermal transition temperatures (T_o_, 92.9 °C; T_p_, 110 °C; and T_c_, 122.4 °C) increased and the enthalpy (ΔH) decreased from 18.2 J/g to 4.3 J/g, indicating the severe disruption of extruded starch (ES) due to the mechanical shear during extrusion [29,30]. Compared with ES, starch extrudates with different moisture contents exhibited similar thermal transition temperatures with a higher enthalpy value. The increased enthalpy may be attributed to the fact that the addition of water enhanced the lubrication effect, reducing the friction among the starch, screw, and barrel in the extruder, which, in turn, reduces the damage to starch granules [13,31,32]. On the other hand, the enthalpy (ΔH) decreased as the moisture contents increased from 30% to 50%, with S-30 showing the highest ΔH (8.3 J/g). Increasing the moisture content can promote the internal mixing of starch, thus enhancing the starch gelatinization [33].

### 3.2. Granular Morphology of Starch and Starch Extrudates

**Figure 2:**
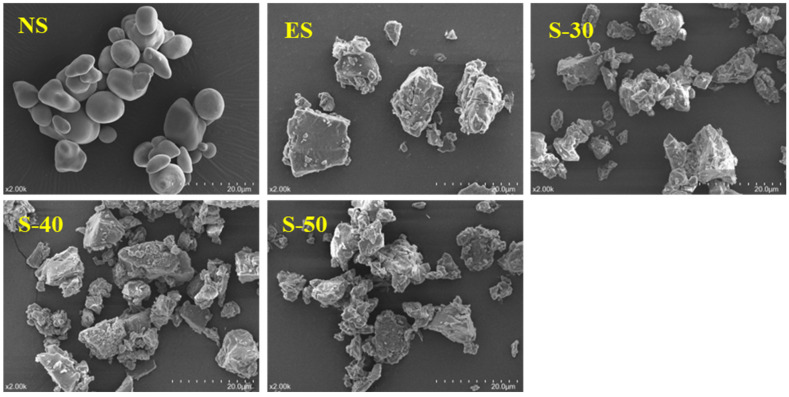
SEM images of high maize 260 (NS) and starch extrudates with different moisture contents (ES, S-30, S-40, and S-50).

The SEM micrographs of starch and starch extrudates are shown in Figure 2. NS showed various shapes from spherical to an elongated rod, consistent with previous research [34]. After extrusion, almost no intact starch granules can be observed, and obvious polygonal chunks with a rough surface can be observed in the ES samples. The starch extrudates with different moisture contents (S-30, S-40, and S-50) exhibited irregular fragment forms, and their morphology did not change significantly with the increasing moisture content. The phenomenon demonstrate that extrusion can largely destroy the structural integrity of starch granules regardless of the moisture contents. The formation of the chunk-structure may be due to the sudden drop in pressure and evaporation of water when the starch material is extruded from the die [35].

### 3.3. Structural Changes of Starch and Starch Extrudates

**Figure 3:**
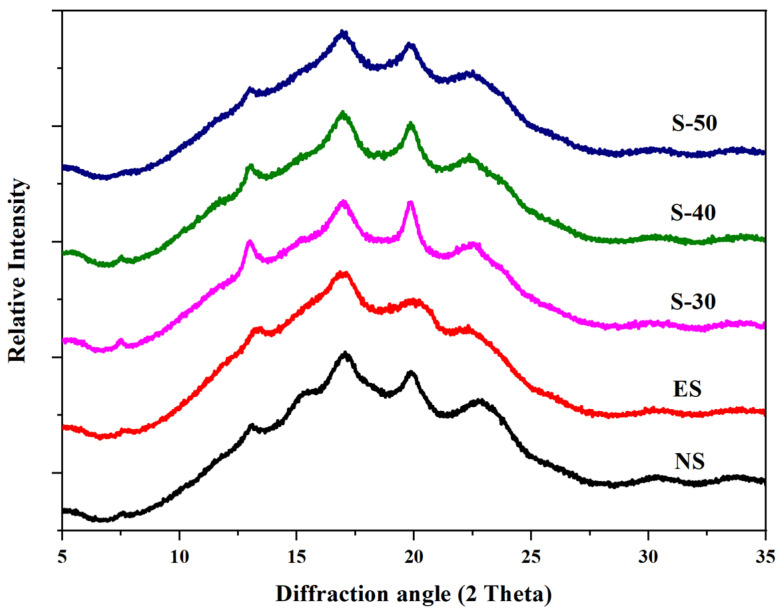
XRD patterns of high maize 260 (NS) and starch extrudates with different moisture contents (ES, S-30, S-40, and S-50).

The X-ray diffraction patterns and relative crystallinities of starch and starch extrudates are shown in Figure 3 and Table 1, respectively. High maize 260 (NS) exhibited a B + V-type crystallinity, with strong reflections at about 7.5°, 13°, 15°, 17°, 20°, and 23° (2θ) [36,37]. The peak at 20° is normally associated with the amylose–lipid complexes. After extrusion, ES retained the signature peaks of B- and V-type polymorphs as NS, but the peak intensity was significantly weakened due to the destructive effect of the shear force on starch during the extrusion process. Correspondingly, the relative crystallinity decreased from 21.7% for NS to 13.3% for ES (Table 1). Notably, starch extrudates with a 30% moisture content (S-30) exhibited a similar B-type and obviously more V-type crystallinity than ES, as evidenced by the stronger peaks around 13° and 20° (2θ) [36]. Particularly, the V-type peaks gradually weaken with the further increase in moisture content (Table 1), consistent with previous studies [38]. It has been reported that a lower moisture content is more conducive to enhancing the availability of amylose chains and promoting the formation of amylose–lipid complexes during the extrusion process of starch [39]. As for the ES sample, no additional water was added, so the mobility of the chains was restricted to a certain extent [40]; thus, the RC value was lower than that of S-30 (Table 1). Moreover, the effects of extrusion on the starch crystalline structure also depends on the starch molecular structures. For the high amylose maize starch in this study, it can still practically retain its crystallinity structure after extrusion, which could be related to its high gelatinization temperature. In contrast, starch with a low amylose content would lose the granular structure associated with the melting of the crystallites and underlying helices, generating a more amorphous structure after extrusion [41].

**Figure 4:**
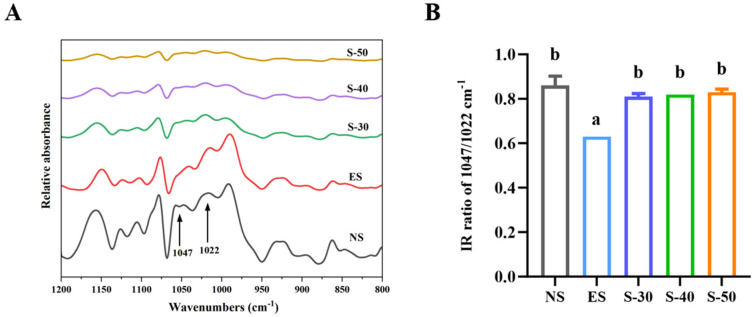
FTIR spectra (**A**) and IR ratio of 1047/1022 cm^−1^ (**B**) of high maize 260 (NS) and starch extrudates with different moisture contents (ES, S-30, S-40, and S-50). Different letters indicate significant differences (*p* < 0.05).

To further investigate the short-range ordered structure of starch during extrusion, the ATR−FTIR spectra were determined and shown in Figure 4. It has been reported that the absorption bands observed between 1300 and 800 cm^−1^ originate from C–O and C–C stretching vibrations and are highly sensitive to changes in the short-range molecular order of starch [42]. The ATR−FTIR spectrum of all samples had the almost same peak position, except starch extrudates with a different moisture content had a lower intensity, differing from NS and ES (Figure 4A). The band at 1047 and 1022 cm^−1^ has been reported to be correlated with the vibrational modes within the crystalline and amorphous phase of starch, respectively [43]. The intensity ratio of 1047/1022 cm^−1^ has been used for estimating the degree of the short-range molecular order in starch molecules [38].

As shown in Figure 4B, the extrusion process of raw starch resulted in a significant reduction in the 1047/1022 cm^−1^ ratio, decreased from 0.86 for NS to 0.63 for ES (Table 1) consistent with the DSC (Figure 1) and XRD (Figure 3) results. Compared with ES, the starch extrudates with higher moisture contents had a higher 1047/1022 cm^−1^ ratio, while there was no difference among S-30, S-40, and S-50. The results suggest that, once the moisture content exceeded 30%, a further increase had little effect on the short-range ordered molecular structure of starch during extrusion.

### 3.4. In Vitro Digestibility of Starch and Starch Extrudates

**Figure 5:**
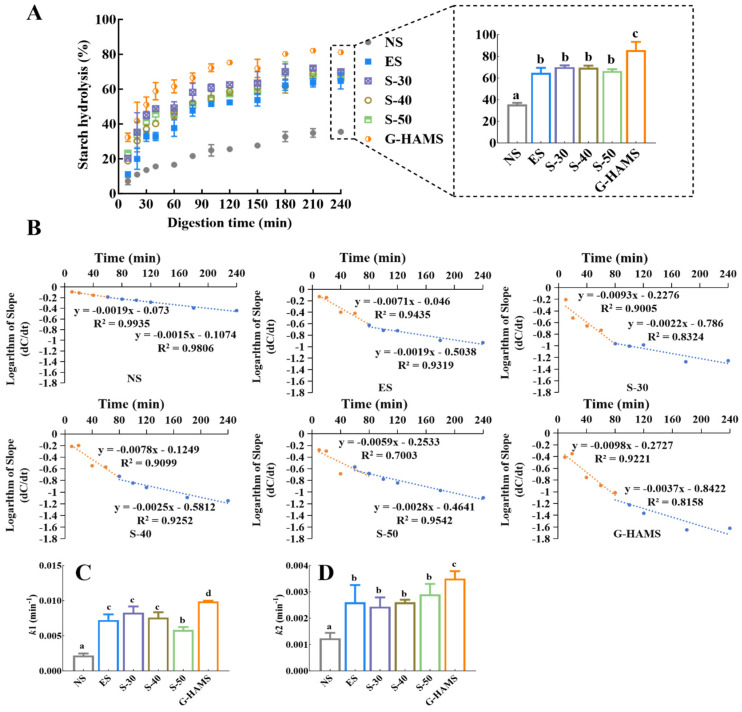
*In vitro* enzymatic digestograms and C240 (%) (**A**), LOS plots (**B**), *k*1 value (**C**), and *k*2 value (**D**) of high maize 260 (NS) and starch extrudates with different moisture contents (ES, S-30, S-40, and S-50). Different letters indicate significant differences (*p* < 0.05).

The *in vitro* enzymatic hydrolysis of starch and starch extrudates are shown in Figure 5, with the digestogram of gelatinized HAMS (G-HAMS) included for reference. The respective digestograms were fitted to the first-order kinetics equation, and LOS plots were used to calculate the digestion rate coefficients, *k*1 and *k*2 (Figure 5B). A gradual increase in starch hydrolysis in NS was observed with the progression. The hydrolysis extent of NS after 240 min of digestion was 36.35%. Compared to NS, extruded starch (ES) and starch extrudates with different moisture contents (S-30, S-40, and S-50) had a significantly higher hydrolysis extent, ranging from 62.17% to 70.90%, with no significant differences between different groups, but all were significantly lower than gelatinized HAMS (81.20%). Compared to NS, the increased digestibility of starch extrudates can mainly be attributed to the following two factors: Firstly, the high temperature and severe shear forces during the extrusion process disrupted the crystalline structure of starch, leading to partial gelatinization. Secondly, compared to the smooth granular surface of NS, the rougher granular surface of starch extrudates can supply more binding sites for digestive enzymes, thus resulting in an increased digestion extent.

As shown in Figure 5B, the LOS plots of all samples showed a two-stage digestion pattern, with NS having the lowest digestion rate constants (*k*1, 0.0019; *k*2, 0.0015). Notably, ES and starch extrudates with different moisture contents exhibited higher digestion rate constants than NS but lower constants than G-HAMS (*k*1, 0.0098; *k*2, 0.0037). Interestingly, compared with S-30 and S-40, S-50 showed a lower digestion rate constant in the rapid digestion phase (*k*1, 0.0059), while, in the slow digestion phase, its digestion rate constant (*k*2, 0.0028) slightly higher than that of starch extrudates (S-30, *k*2, 0.0022; S-40, *k*2, 0.0025).

### 3.5. In Vitro Fecal Fermentation Characteristics of Starch and Starch Extrudates

#### 3.5.1. Gas Production and pH Changes

**Figure 6:**
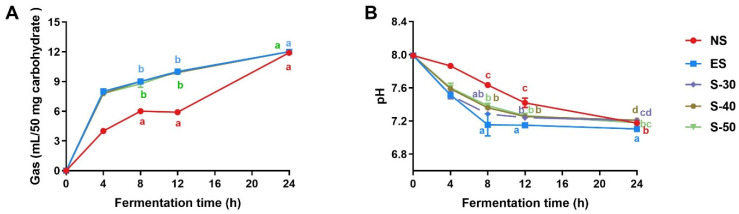
Gas production (**A**) and pH change profiles (**B**) during 24 h *in vitro* fecal fermentation of high maize 260 (NS) and starch extrudates with different moisture contents (ES, S-30, S-40, and S-50). Different letters indicate significant (*p* < 0.05) differences at the same fermentation time.

It is generally believed that the production of gas is a rough indicator for the fermentation rate of dietary fibers [44]. The gas production of starch and starch extrudates are shown in Figure 6A. NS generated the lowest gas production in the initial 12 h and presented a nearly linear gas production profile from 12–24 h of fermentation; notably, its gas production was comparable to that of ES at the end of fermentation. After extrusion, ES and starch extrudates with different moisture contents (S-30, S-40, and S-50) exhibited significantly higher gas production in comparison with NS throughout the whole fermentation period. Notably, the gas production profiles of starch extrudates with different moisture contents almost overlapped during the fermentation process, indicating that the moisture content is not the main factor affecting the fermentation rate during extrusion.

The changes in the pH value during the *in vitro* fermentation of starch and starch extrudates are shown in Figure 6B. The pH levels of the fermentation culture were 7.99 in various groups. Consistent with the gas results, NS showed the slowest pH decrease rate among all the groups throughout the whole fermentation period. In contrast, the fermentation of ES resulted in significantly lower pH values at each time point compared with those of NS. It was noteworthy that starch extrudates with different moisture contents (S-30, S-40, and S-50) exhibited higher pH values than those of ES at 8–24 h of fermentation, while there was no difference in pH values among groups with different moisture contents.

#### 3.5.2. Short-Chain Fatty Acids Analysis

**Figure 7:**
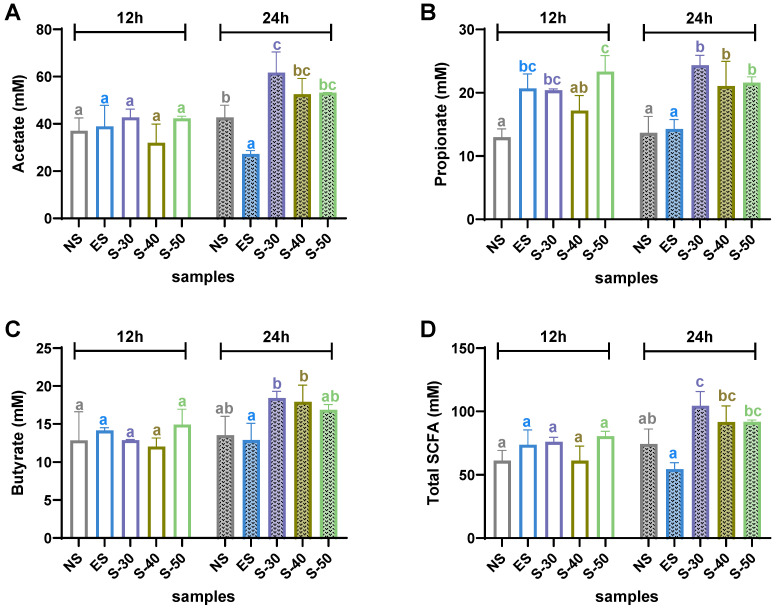
Acetate (**A**), propionate (**B**), butyrate (**C**), and total SCFA (**D**) production at 12 h and 24 h *in vitro* fecal fermentation of high maize 260 (NS) and starch extrudates with different moisture contents (ES, S-30, S-40, and S-50). Different letters indicate significant (*p* < 0.05) differences at the same fermentation time.

To further examine the metabolites, SCFAs including acetate, propionate, butyrate, and total SCFA production at 12 h and 24 h of fermentation were determined and presented in Figure 7. After 12 h of fermentation, ES and starch extrudates with different moisture contents (S-30, S-40, and S-50) exhibited higher propionate production compared to NS, while no significant differences were found in the production of acetate, butyrate, and total SCFA. After 24 h of fermentation, the production of propionate and butyrate in ES was comparable to that in ES, while the production of acetate decreased significantly. Compared with ES, starch extrudates with different moisture contents (S30, S-40, and S-50) displayed significantly higher concentrations of acetate, propionate, butyrate, and total SCFA. Notably, there were no significant differences in the acid production among the starch extrudates with different moisture contents.

#### 3.5.3. Pearson Correlation Analysis

To evaluate the impact of the moisture content on the structure and functional properties of starch, starch extrudates with varying moisture contents (30%, 40%, and 50%) were prepared by a twin-screw extrusion, with starch extrudates without an additional water addition (NS) as the control group. The structural and morphology changes were assessed by DSC, XRD, FTIR, and SEM, respectively. The digestibility in the small intestine and colonic fermentability were determined through *in vitro* mimic intestinal digestion experiments and an *in vitro* fecal fermentation model, respectively. The results indicate that, compared to NS, the structure in ES was significantly disrupted after extrusion due to the shear forces generated during the screw extrusion process [29]. Compared to ES, the addition of water reduces the starch damage extent to some extent, as the water acts as a plasticizer during extrusion, and reduced the friction between the starch, screw, and barrel of the extruder [31,45]. On the other hand, the gelatinization degree increased with the increasing moisture contents. However, there were no significant differences in the digestion extent, fermentation rate, or SCFA yields among starch extrudates with different moisture contents. To gain a better understanding of the relationships between the structure and function of starch and starch extrudates, Pearson correlation analysis was determined and shown in Table 2. Obviously, *k*1 and *k*2 were negatively correlated with the ΔH, IR ratio of 1047/1022 cm^−1^, and RC, suggesting both the long- and short-range order of the starch structure can slow down the starch digestion rate [46,47]. Notably, there were significant correlations between RC (V-type) and the production of acetate (r = 0.93, *p* < 0.01), propionate (r = 0.88, *p* < 0.05), and butyrate (r = 0.90, *p* < 0.05), indicating the V-type starch complex can promote the production of SCFAs. The V-type starch complex has been reported to be more conducive to the production of SCFAs, possibly because the V-type structure provides substrate specificity for microorganisms in the colon that selectively produce SCFA [48,49,50]. The correlation analyses also provide evidence to support the higher SCFA production in starch extrudates with different moisture contents (S-30, S-40, and S-50) than those in ES.

## 4. Conclusions

In conclusion, the extrusion processing severely disrupts both the long- and short-range ordered structures of high amylose maize starch, increases the amylase sensitivity, and simultaneously accelerates the *in vitro* fermentation rate. Compared to ES, the water addition mitigated the structural disruption, resulting in the formation of more of the V-type starch complex, with the highest content of the V-type complex in S-30. Although long-range ordered structures of starch extrudates decreased with increasing moisture content, digestibility and fermentation rates remained comparable across treatments. Notably, compared to ES, starch extrudates with different moisture contents produced significantly higher SCFAs concentrations after 24 h of fermentation, which may be related to the formation of the V-type starch complex. These findings provide a better understanding of how the moisture content during extrusion affects the digestibility of the starch extrudates as well as the fermentation metabolites. Furthermore, how V-type starch complexes promote the production of short-chain fatty acid is also of interest for further studies.

## Figures and Tables

**Table 1:** Thermal properties, relative crystallinity, and infrared (IR) ratios of high maize 260 (NS) and starch extrudates with different moisture contents (ES, S-30, S-40, and S-50).

Sample	T_o_ (°C)	T_p_ (°C)	T_c_ (°C)	ΔH (J/g)	IR Ratio of 1047/1022 cm^−1^	RC (B+V-Type) (%)	RC (V-Type) (%)
NS	88.0 ± 1.4 a	101.9 ± 2.3 a	120.9 ± 0.6 a	18.2 ± 0.2 d	0.86 ± 0.03 b	21.7	6.2
ES	92.9 ± 1.0 b	110.0 ± 5.4 ab	122.4 ± 1.4 a	4.3 ± 0.2 a	0.63 ± 0.00 a	13.3	5.7
S-30	92.4 ± 0.6 b	109.3 ± 0.5 ab	119.2 ± 1.9 a	8.3 ± 0.3 c	0.81 ± 0.01 b	15.1	7.6
S-40	93.6 ± 0.3 b	110.6 ± 0.6 ab	125.4 ± 1.8 a	6.0 ± 0.3 b	0.82 ± 0.00 b	13.2	6.8
S-50	93.3 ± 0.1 b	114.2 ± 0.8 b	123.9 ± 1.0 a	5.9 ± 0.2 b	0.83 ± 0.01 b	13.1	6.5

Values are means ± SD. Significant (*p* < 0.05) differences between microspheres are indicated with different letters. RC: relative crystallinity; IR ratio of 1047/1022 cm^−1^: the infrared ratio of absorbances at 1047/1022 cm^−1^.

**Table 2:** Pearson correlation coefficients among structural characteristics, digestion kinetic parameters, and SCFA production of high maize 260 (NS) and starch extrudates with different moisture contents (ES, S-30, S-40, and S-50).

	ΔH	IR Ratio of 1047/1022 cm^−1^	Total RC	RC of V-Type	*k*1	*k*2	Acetate	Propionate	Butyrate
ΔH	1								
IR ratio of 1047/1022 cm^−1^	0.57	1							
Total RC	0.99 **	0.46	1						
RC of V-type	0.26	0.79	0.162	1					
*k*1	−0.88	−0.35	−0.87	0.45	1				
*k*2	−0.95 *	−0.52	−0.94 *	0.13	0.69	1			
Acetate	0.03	0.74	0.51	0.93 **	0.30	−0.10	1		
Propionate	−0.42	0.38	−0.40	0.88 *	0.68	0.33	0.89 *	1	
Butyrate	−0.32	0.45	−0.30	0.90 *	0.63	0.20	0.92 *	0.97 **	1

ΔH, enthalpy change; IR ratio of 1047/1022 cm^−1^, the ratios of absorbances at 1047/1022 cm^−1^; RC, relative crystallinity; *k*1, the first-order rate coefficient; *k*2, the second-order rate coefficient; Acetate, acetate production; Propionate, propionate production; Butyrate, butyrate production. Significant correlations are indicated in bold, with * *p* < 0.05 and ** *p* < 0.01.

## Data Availability

The original contributions presented in this study are included in the article. Further inquiries can be directed to the corresponding author.
